# Irrelevance of *USF2* rs916145 polymorphism with the risk of biliary atresia susceptibility in Southern Chinese children

**DOI:** 10.1042/BSR20193623

**Published:** 2020-02-28

**Authors:** Lei Chen, Ming Fu, Ledong Tan, Jinglu Zhao, Xiaogang Xu, Yuzhen Lin, Qian Zhong, Ruisui Zhong, RuiZhong Zhang, Jixiao Zeng

**Affiliations:** Department of Pediatric Surgery, Guangzhou Institute of Pediatrics, Guangzhou Women and Children’s Medical Center, Guangzhou Medical University, Guangzhou, China

**Keywords:** Biliary Atresia, Case-control, Polymorphism, Risk, USF2

## Abstract

**Backgrounds:** Biliary atresia (BA) is a very rare neonatal disease, however, it has been the most common cause of obstructive jaundice in infancy. The complex pathogenesis of BA is not entirely clear and a lot of possible pathogenic mechanisms have been proposed to explain the etiology of BA, including genetic, inflammatory, environmental and developmental abnormalities. As a transcription factor, *USF2* gene rs916145 polymorphism has been shown to be related to the risk of BA.

**Methods:** We examined the *USF2* rs916145 genotype in a large case–control study consisting of 506 BA patients and 1473 healthy controls, using the MassARRAY iPLEX Gold system (Sequenom). Odds ratios (ORs) and 95% confidence intervals (CIs) were used to evaluate the association between the *USF2* gene rs916145 polymorphism and BA susceptibility.

**Results:** The frequency of different genotypes showed no statistical significance (GG/GC, OR: 1.09, *P*=0.470, 95% CI: 0.87–1.35; GG/CC, OR: 0.86, *P*=0.378, 95% CI: 0.62–1.20). No obvious association was revealed between the *USF2* gene rs916145 polymorphism and BA susceptibility.

**Conclusion:** *USF2* rs916145 polymorphism may not be the best predictor of BA.

## Introduction

Biliary atresia (BA) is a very rare neonatal disease, although this is the most common cause of obstructive jaundice in infants. The complex pathogenesis of BA is not entirely clear, for which high quality is still limited to a few studies from specialized centers [1]. BA manifests itself as the obliteration of the extrahepatic bile ducts, disrupting bile flow [2]. The incidence of BA shows various results in different racial groups, which is more common in Asian populations than that in West Europe (approximately 1/5000 Asians vs 1/18000 whites) [3]. So far, the first choice for improving the short-term outcome in BA children is hepatoportoenterostomy (HPE) after surgical removal of duct remnants, although most of them will progress to end-stage cirrhosis, eventually requiring liver transplant by adulthood [4,5]. Changing the poor perception of BA pathogenesis is a huge challenge as well as a hope for improvements of BA prognosis.

The etiology of BA is still uncertain, and a lot of possible pathogenic mechanisms have been proposed to explain the etiology of BA, including genetic, inflammatory, environmental and developmental abnormalities [6–8]. Several authors have suggested that a number of genes showed association with BA, such as *CD14* [9], *CFC1* [10], migration inhibitory factor (*MIF*) [11] and *IL18* [12]. Gene mutations were deemed to have links to BA, however, remaining unclear. Growing interests are garnered in the studies exploring gene sequence variants as susceptibility factors for BA, such as the single nucleotide polymorphisms (SNPs) including *ITGB2, ADIPOQ* and *VEGF* [13–15].

USF2, one of the upstream stimulatory factors (USFs) with another one named USF1, belongs to the basic helix–loop–helix leucine zipper family of transcription factors [16]. The target genes of USF2 always share a common feature that within their promoters USF2 can bind as homodimers or heterodimers to E-boxes with a 5′-CANNTG-3′ DNA-core sequence [17,18]. With the ability to act as either transcriptional activators or transcriptional repressors of various genes, USF2 is recognized to play crucial roles in the control of growth and developmental process as it has been shown to affect embryogenesis, fertility and growth, brain function and iron homeostasis [17,19–21]. In addition, several studies have also been conducted to verify the relationship between *USF2* polymorphisms and various diseases. Shibata et al. [22] reported that the SNPs of *USF2* gene did not show significant association with onset of Alzheimer’s disease (AD). However, another study carried out in 52 patients of BA and 96 healthy controls for *USF2* gene SNPs (rs7251432 and rs916145) showed that C allele of rs916145 in *USF2* gene had more frequency for developing BA [23].

With the purpose to further evaluate the association of *USF2* with BA, we designed a case–control study to verify the relationship of rs916145 in *USF2* gene with BA susceptibility in a Southern Chinese children population, consisting of 506 BA patients and 1473 healthy controls.

## Materials and methods

### Study subjects

Chinese subjects consisting of 506 BA patients and 1473 healthy controls were all recruited at the Guangzhou Women and Children’s Medical Center in the present study, with a mean age being 2.088 ± 1.934 months (range: 1–7 months). The cases were all tested by ultrasonography evaluation before surgical operations and they were all diagnosed by intraoperation cholangiography and confirmed by pathology after surgery. A total of 1473 healthy children were randomly selected as the controls who had no history of liver or autoimmune disease, just aiming to receive a routine physical examination in the same hospital. The present study has been approved by the Institutional Review Board of Guangzhou Women and Children’s Medical Center and all participants or their guardians have signed informed consent.

### Polymorphism analysis

Genomic DNA was extracted from peripheral blood samples (approximately 2 ml) by the usage of the TIANamp Blood DNA Kit (TianGen Biotech, Beijing, China) according to the manufacturer’s instructions. Then, a UV spectrophotometer (NanoDrop Technologies, Wilmington, DE) was used to determine DNA concentration and purity by measuring spectrophotometer absorbance at 260 and 280 nm. The SNP rs916145 of *USF2* gene was selected from the previous study conducted by Huang et al. [23] and successfully designed using the MassARRAY iPLEX Gold System (Sequenom). The TaqMan™ SNP Genotyping Assay of USF2 rs916145 whose context sequence is ‘GTAAGCTTGCTCTGGAGAGGATGTA[C/G]CTGCAGCCGGCGCCCAGCTCTCGAG’ was bought from Thermo Fisher (catalog# 4351379, Applied Biosystems™). DNA samples were qualified and diluted to 10 ng/μl and loaded in 96-well plates for the analysis. The Hardy–Weinberg equilibrium showed significant deviation in all controls, passing the test with *P*-value >0.05 (*P*=0.965). In addition, we performed replication assays randomly in 5% of the samples and the results were 100% concordant.

### Statistical analysis

All statistical analyses were conducted by using SAS software (version 9.4, SAS Institute, NC, U.S.A.). Two-sided χ^2^ tests were used to analyze the demographic data and genotype frequencies. The Hardy–Weinberg equilibrium was assessed by goodness-of-χ^2^ test in healthy controls. Odds ratio (OR) and 95% confidence interval (CI) were calculated to evaluate associations between *USF2* polymorphisms and BA susceptibility. OR and 95% CI were also estimated in dominant and recessive models. All tests were two-sided, and *P*<0.05 was considered statistically significant.

## Results

### Association of *USF2* SNP with BA susceptibility

In the present study, the SNP rs916145 of *USF2* gene was examined in 506 cases and 1473 controls, which was consistent with HWE (HWE = 0.965) in the healthy controls. But we failed to find any significant association between rs916145 with risk of BA in individual genotype analysis from the results shown in Table 1. The frequency of different genotypes (GG, GC, CC) in cases (38.07, 50.00, 11.93%) was basically equivalent to that in controls (38.83, 47.01, 14.16%). Likewise, the analysis within dominant model and recessive model also showed no positive results. To sum up, based on the results in the present study, we could not detect the obvious association of *USF2* rs916145 polymorphism with the susceptibility of BA.

**Table 1:** Genotype distributions of rs916145 G>C polymorphism and BA risk

Genotype	Cases (*n*=506)	Controls (*n*=1473)	*P*^1^	Crude OR (95% CI)	*P*	Adjusted OR (95% CI)^2^	*P*^2^
**rs916145 G>C** (HWE = 0.965)							
GG	185 (38.07)	565 (38.83)		1.00		1.00	
GC	243 (50.00)	684 (47.01)		1.09 (0.87–1.35)	0.470	1.10 (0.88–1.37)	0.419
CC	58 (11.93)	206 (14.16)		0.86 (0.62–1.20)	0.378	0.86 (0.62–1.21)	0.387
Additive			0.682	0.97 (0.83–1.13)	0.682	0.97 (0.84–1.13)	0.715
Dominant	301 (61.93)	890 (61.17)	0.764	1.03 (0.84–1.28)	0.765	1.04 (0.84–1.29)	0.708
Recessive	428 (88.07)	1249 (85.84)	0.216	0.82 (0.60–1.12)	0.216	0.82 (0.60–1.12)	0.211

1 χ^2^ test for genotype distribution between BA patients and controls.

2 Adjusted for age and gender.

## Discussion

As the most common serious pediatric liver disease of infancy, BA has complex genetic etiology and the underlying cause(s) and outcome contributor(s) still remain largely unknown [24]. With the application of new genome technologies which contributes to promote the discovery of novel disease-causing genes in BA. Genome-wide association studies (GWAS) that investigate the association of SNPs with diseases are usually taken to inquire the genetic underpinning of BA. Through a previous GWAS on Han Chinese, Cheng et al. [25] discovered the correlation of the 10q24.2 region encompassing *ADD3* and *XPNPEP1* genes, which was confirmed in Chinese and Thai populations. Our earlier study also revealed the intragenic epistatic association of *ADD3* with BA in Southern Han Chinese population [26].

In the present study, we evaluated the replication results of *USF2* rs916145 polymorphism in a larger case–control population with 506 cases and 1473 controls, compared with the same study conducted by Huang et al. [23] in 2008, which demonstrated that C allele and CC allele of rs916145 in *USF2* gene had more frequency for developing BA in 52 BA patients and 96 healthy controls. However, in our study, we found that the rs916145 genotype (CC, GC, GG) had the similar frequency in 506 BA patients (11.93, 50.00, 38.07%) and 1473 normal controls (14.16, 47.01, 38.83%), giving limited association between rs916145 polymorphism and risk of BA. The analysis using different gene models (dominant and recessive models) also showed no obvious linking for rs916145 polymorphism to BA. The allele frequency was slightly different between the two studies, which could be due to the limit of previous study’s sample size or potential racial stratification.

Something ought to be noticed here is that several limits still exist in the present study, such as the environmental factors that have not been identified yet. In future study designs, age at HPE, environmental toxins and evidence of cytomegalovirus infection should be considered when establishing the inclusion and exclusion criteria [27,28]. In consideration of the low incidence and underlying racial effect of BA, larger replication in an independent cohort involving different ethnicities was still required.

To sum up, our study shows that *USF2* gene SNP rs916145 has no significant correlation with the risk of BA in the Southern Chinese children population. *USF2* rs916145 polymorphism may not be the best predictor of BA.

## Abbreviations

BA: biliary atresia
CI: confidence interval
GWAS: genome-wide association study
HPE: hepatoportoenterostomy
OR: odds ratio
SNP: single nucleotide polymorphism
